# The Physio-Psychological Effect of Forest Therapy Programs on Juvenile Probationers

**DOI:** 10.3390/ijerph18105467

**Published:** 2021-05-20

**Authors:** Jin Young Jeon, In Ok Kim, Poung-sik Yeon, Won Sop Shin

**Affiliations:** 1 Department of Forest Therapy, Chungbuk National University, Cheongju 28644, Korea; forest-bb@naver.com (J.Y.J.); inoya88@hanmail.net (I.O.K.); 2 Department of Forest Sciences, Chungbuk National University, Cheongju 28644, Korea; imoscow@hanmail.net

**Keywords:** juvenile delinquents, adolescents, forest therapy, anti-recidivism programs, psychological well-being, HRV

## Abstract

The study aimed to investigate the psychological and physiological effects of forest therapy programs on adolescents under probation. Fifty probationary teenagers from the Ministry of Gyeonggi Justice Compliance Support Center participated in the study. The study explored the effectiveness of a nonrandomized control group pretest–posttest design forest therapy program. The forest therapy program was conducted for two days and one night for the experimental groups (N = 33), who participated in the forest therapy program, and the control group (N = 17), who received two days of attendance center orders program in the lecture room of the Ministry of Gyeonggi Justice Compliance Support Center. As a result, adolescents under probation who participated in forest therapy programs had a beneficial effect on psychological well-being (K-WBMMS) and HRV’s HF (high frequency) and LF/HF (A ratio of Low Frequency to High Frequency) compared to those who received the general attendance center orders program. These results support that forest therapy programs play a positive role in the psychological and physiological effects of probationary adolescents and can affect the diversity of rehabilitation programs for probationary adolescents.

## 1. Introduction

Delinquency, which was previously limited to unsupervised wandering, has emerged as a serious social problem as the proportion of violent crimes such as theft, school violence, group sexual assault, and murder has increased daily. Since 1989, South Korea has implemented a probation system that improves criminality in accordance with volunteer activities and attendance center orders, while living a normal life at home, school, and the workplace, and without imprisoning teenagers who commit crimes [1]. In the case of juvenile crimes, the premise is that these individuals are not fully developed when compared to adults; therefore, delinquency is managed through protection and education, rather than strong punishment [2].

Youth crime rates continue to decline due to the efforts of programs for adolescents under probation. In 2018, the number of probationary teenagers stood at 35,626, 4.2% less than that in the previous year. Also, the proportion of teenagers who received their first probation in the past decade accounted for 48.3% in 2018, compared to 66.1% in 2009 [3]. However, unlike the declining trend of probationary teenagers, there remains work to be done regarding juvenile delinquency. According to the crime white paper of the Ministry of Justice [4], the proportion of crimes by women is increasing based on the status of probation in 2018, and 16- to 17-year-olds account for the highest percentage of all criminal and special law crimes. The highest numbers of charges were for theft (11,625; 34.9%) and violence (4207; 12.6%). In particular, the biggest problem is that approximately 90% of all repeat offences occur within a year. In 2018, Korea’s crime rate for probationary teenagers was 15.7% within one month, 24.1% within three months (one to three months), 23.4% within six months (three to six months), and 26.4% within a year (six months to one year), with a total crime rate of 89.6%. This shows that the target number of probationary teenagers is much higher than that of adults. To solve these problems, an important strategy involves improving the attendance center order programs for probationary youth.

Adolescence is the stage of life in which people experience the most risks, opportunities, frustrations, and achievements [5]. According to Ryff [6], psychological well-being is the sum of psychological aspects constituting an individual’s quality of life. People with a high quality of life accept themselves as they are and maintain positive interpersonal relationships. They can control their behavior, surrounding, sense of purpose, and motivation to reveal their potential [7]. Armsden and Greenberg [8] reported adolescents with healthy attachments to parents and peers reported higher self-esteem and life satisfaction; occurrences of depression, anxiety, guilt, anger, alienation, and self-concept confusion were lower. Kim et al. [9] reported that these relationships add to the experience of school life, as good relationships are related to high satisfaction with school. Psychological well-being in adolescence allows young people to grow and form their judgments independently. Receiving good social support from relationships with family and peers are important factors that affect personal development and psychological health [10,11,12].

However, compared to adolescents who come from stable environments, juvenile offenders usually hail from vulnerable environments, where their psychological, familial, peer group and social resources are insufficient for healthy growth and development. Difficulties in emotional support due to specific family structures such as single parents, divorce, or remarriage [13,14,15], inconsistent parenting [16,17], deviant peer on delinquency [18], depression [19,20], low self-concept [21], impulsiveness, and aggression [22,23,24] are factors that increase juvenile delinquency. It is expected that criminals in this situation experience low levels of happiness and high levels of psychological anxiety. The stigma of being labeled a criminal is a stressor for juvenile offenders and is related to their depression and low self-esteem [25,26]. Low self-esteem is a powerful influencer of violent behavior [27]. According to Lim et al. [28], the negative emotional experience of juvenile offenders affects impulsive behavior and aggression, which directly affects delinquency. If juvenile delinquents fail to properly deal with stress reactions such as depression, dissatisfaction, social contraction, and aggression, they will show problems such as drinking, drug use, running away from home, suicide, and smoking [29,30]. The continuation of this life can be said to affect the stress control of juvenile offenders.

The influence of stress may vary depending on how stress coping resources control stress [31]. High levels of stress coping resources in stressful situations can buffer the negative effects of stress. However, juvenile delinquents who have lower stress response capabilities than ordinary teenagers are bound to be poor at coping with stress, which can negatively affect even physical reactions. Stress and physical symptoms showed a static correlation [32,33]. Stress reactions can be identified through autonomic neuronal activity. In situations such as negative emotions or stress imposition, sympathetic nerve activity increases, and parasympathetic nerves are activated when positive emotions are triggered. If a stressful situation persists, it is difficult to maintain a balanced life between mind and body. Therefore, it is necessary to properly manage the emotional tension of juvenile delinquents to relieve stress-induced physical tension.

However, the current attendance center orders require a professional program that deals with emotional and physical stability as much as retraining to prevent re-offending and help young probationers develop healthy growth in the form of group therapy such as mental development training, human relationships, sex education, and substance abuse education [34]. To this end, the Ministry of Justice actively links government ministries and private resources to promote more effective delinquent behavior correction and prevention of recidivism through experiential learning, volunteer work, cultural arts programs, etc. Recently, a program has been developed to help the unstable situation of probationary youth considering their characteristics [35,36,37,38]. Attempts by various programs, such as mentoring, art therapy, and meditation, have shown effects such as stabilizing emotions, reducing impulsiveness and aggression, and improving empathy among teenagers, and as a result, factors affecting delinquent behavior have improved.

According to a report by Lee et al [39], subjective evaluations of probationary adolescents were conducted on the attendance center orders program. As a result, leisure culture and hobby-based activities were most helpful in reducing delinquency behavior and stabilizing psychology and emotions. Motivation for participation was also found to have a higher preference for dynamic activities centered on experience or play than lecture ceremonies. Probationary teenagers are being treated in the form of internal treatment. The need to develop various programs aimed at recovering risk factors for youth, such as self-esteem, sociality, and mental balance, is constantly mentioned to overcome difficulties in daily life and to return to society smoothly.

Interest in forest activity intervention is increasing day by day to help teenagers participate in effective attendance center order programs. Forest activities are known to play a positive role as a space for psychological and physical recovery. It has been reported that forest activities relieve negative emotions, such as depression and anger [40,41,42] and provide stress recovery [43,44,45]. These positive psychological effects create positive physiological effects, such as parasympathetic nerve activation [42,46,47,48,49] and stress hormone reduction [49,50]. In addition to solely experiencing the forest, research on the effectiveness of mental and physical health promotion activities is also underway through a program that is systematically organized using the sounds, scents, landscapes, and natural objects of the forest. This is also applicable to forest therapy programs for teenagers.

Forest therapy programs use physical activities and psychotherapy to help improve the health of adolescents. Forest activities can give physical energy to teenagers. Through various activities using natural objects, teenagers experience a sense of achievement, fun, and immersion, and improve their concentration. Through this, teenagers’ confidence and personal capabilities can be increased, and their interpersonal capabilities can be enhanced through cooperation and intimacy with the members who worked together [51]. According to Chang et al. [52], forest experience programs have been shown to help adolescents adapt and cope by reducing depression and anxiety and positively impacting their self-concept. Cho et al. [53] conducted forest education programs in spring, summer, and autumn and showed improvements in adolescents’ psychological well-being and reduction of stress levels, regardless of the season. Forest education programs also appear to effect physiological changes. Lee [54] studied the impact of Natural Park experiences on heart rate variability (HRV) and found that parasympathetic nerve activity was activated and sympathetic nerve activity, which is activated under stress, was inhibited. Furthermore, Chung et al. [55] studied a three-night and four-day forest therapy camp, considered to be a special group, and found changes in resilience, interpersonal relationships, and heart rate intervals. It was revealed that the forest experience not only had a positive impact on adolescents’ development of social sentiments, such as emotional acceptability and improved emotional control, but also provided physiological stability.

Studies of juvenile offenders with forest experience also showed potential psychological healing [56,57,58,59,60]. A study by Eom et al. [56] reported that a two-day forest education program had a positive effect on the mood, self-esteem, and self-control of probationary youth. Jang et al. [57] reported that forest education programs showed positive changes in self-esteem and resilience. Walsh [58] reported that program activities in nature have shown great changes in young offenders’ hopes, resilience, and self-efficacy.

These previous studies show that forest therapy helps develop prosocial connections and bonds and introduces positive psychological and physiological stimuli for juvenile offenders. However, there is still a lack of underlying research to help young people with psychological and physical stability through forest therapy programs, and more evidence on health benefits is needed. Therefore, this study aimed to investigate whether forest therapy developed by referring to the characteristics of probationary adolescents affected their psychological and physical conditions.

**Hypothesis** **1.** *Adolescents under probation who have experienced forest therapy programs will see a greater positive change in their psychological well-being in comparison to those who have attendance center orders.*

**Hypothesis** **2.** *Adolescents under probation who have experienced forest therapy programs will show a greater positive change in HRV than those who have attendance center orders.*

## 2. Materials and Methods

### 2.1. Participants

The program was conducted with 50 juvenile probationers from the Gyeonggi Justice Compliance Support Center. This study was conducted with the approval of the IRB (CBNU-201809-SB-711-01) of Chungbuk National University Industry–Academic Cooperation Foundation. The researcher made a recruitment announcement to a Justice Compliance Support Center in Korea to conduct a forest therapy program for probationary youth. After a meeting with a Justice Compliance Support Center that expressed its willingness to participate in the program and the overall progress of the program, the probationary youth were notified of the recruitment. Participants were selected from those who completed a voluntary participation agreement after explaining the purpose and content of the study. The forest therapy program was conducted in different groups of participants, and in the case of the experimental group, the two-day, one-night program was divided into 8 people in the first, 9 people in the second, and 13 people in the third. The control group had 17 participants in two days.

Most of the juvenile probationers who participated in the study identified as “male” (92.5%). The age range was 15 to 20 years old. The average age of the experimental group was 16.4 years, and of the control group was 15.8 years. Participants included 14 middle school students (experimental group *N* = 8, control group *N* = 6) and 36 high school students (experimental group *N* = 25, control group *N* = 11). An overview of the demographic data is shown in Table 1.

### 2.2. Experimental Sites

**Figure 1:**
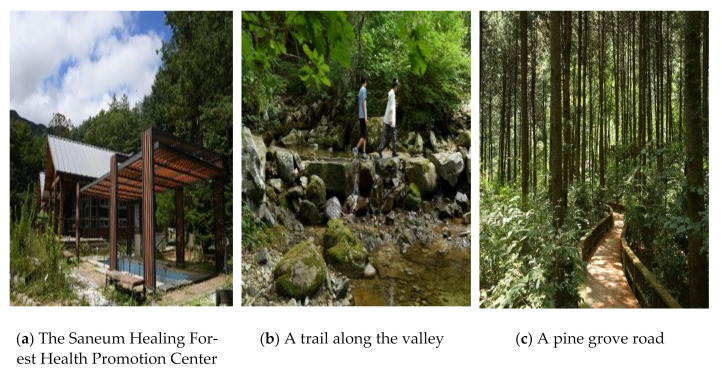
The Saneum Healing Forest.

The Saneum Healing Forest is the first healing forest in Korea. It is composed of various tree species, such as pine (*Pinus Densiflora Siebold &* *Zucc*), larch (*larix kaempferi (Lamb.) Carrière*), and ash (*fraxinus rhynchophylla*), among others. The natural environment is excellent. The altitude of Saneum Healing Forest varies from about 200 to 1000 m, making it suitable for forest therapy programs using climate and exercise therapy. The valley, which has abundant water resources, is located around the Health Promotion Center. By generating anions, Healing Forest Road is known to make people feel increased immune health, mental stability, and freshness. The Saneum Healing Forest is a suitable destination for forest therapy based in sensory programs (see Figure 1).

### 2.3. Procedure

The forest therapy program ran from August to September 2018. The study explored the effectiveness of a non-randomized control group pretest–post-test design forest therapy program. The forest therapy program was conducted for two days and one night for the experimental groups (N = 33), who participated in the forest therapy program, and the control group (N = 17), who received two days of an attendance center orders program. A pretest measurement was conducted before the forest therapy program began. On the first day, participants arrived at the Healing Forest Center to understand the questionnaire, fill out the psychological well-being questionnaire, and measure their HRV. After the pretest, the schedule for the next two days and one night was introduced, with precautions for safety. Participants placed their luggage in their accommodations, finished their lunches, took a break, and began the forest therapy program. After the first day of the program, they stayed overnight at the Saneum Recreation Forest and proceeded with the program the next morning. After sharing their impressions, the program ended. The questionnaires were filled in and HRV was measured. After these were completed, the participants returned home with a probation officer.

**Figure 2:**
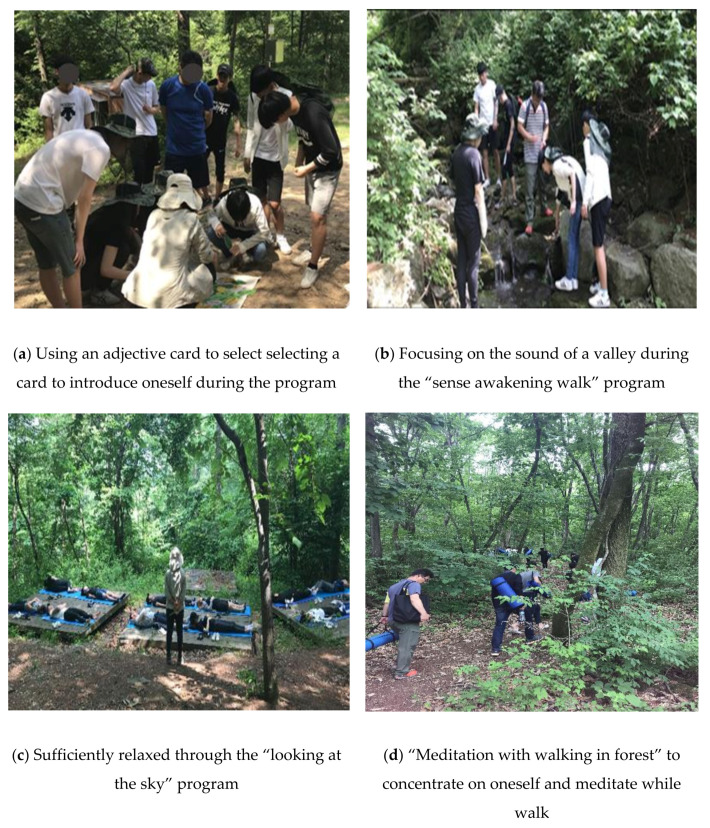
Major progress of forest therapy program.

The program consisted of three themes, and its purpose was to observe positive psychological and physical changes through activities in the forest. The first theme, “Go to the Forest” (“body, hello!”, “introduce myself using natural objects”), was designed to raise interest in forests, form intimacy between participants, and prepare participants to adapt to the new environment. The second theme, “Do in the Forest” (“sense awakening walk”, “dream of trees”, “my dream”, “meditation with walking in forest: slow pace”, “looking at the sky”, and “night walk in the forest”) aimed at relaxing the mind and body through walking in the forest and meditating in the forest environment through five senses. Moreover, they were encouraged to reflect on the past and think about the future. The final theme, “With the Forest and Well” (“storytelling: my dream”, “phytoncide breathing meditation”, “hammock healing”, “scent of forest therapy), was designed to provide an opportunity to define dreams and visions for the future (see Figure 2).

The forest therapy program was conducted by two forest therapy instructors. Two probation officers participated in the program to encourage lagging participants. They participated in the play to increase the participants’ immersion and to safeguard the participants. Each program’s progress time and unit program were adjusted according to the participant’s immersion or condition. Detailed forest therapy program activities were scheduled as shown in Table 2. Participants in the control group visited the Ministry of Justice Compliance support center for two days and were required to carry out the attendance center orders program. Pre- and post-measurements were performed in the same way as the experimental group. The control group was instructed to continue general life routines, except for visiting the natural environment.

### 2.4. Measurement

#### 2.4.1. Psychological Well-Being (Well-Being Manifestation Measure Scale)

To measure the psychological well-being of adolescents, Park and Choi [61] translated and calibrated into Korean the Well-Being Manifestation Measure Scale developed by Masse et al. [62]. The subfactor of this scale is the five-point Likert scale (1 = strongly disagree; 5 = strongly agree), consisting of a total of six subfactors and 25 questions measuring self-esteem, mental balance, sociability, social involvement, control of self and events, and happiness. The higher the score of each question, the higher the level of psychological well-being of adolescents. The Cronbach’s α of the original measurement was 0.92.

#### 2.4.2. HRV

The HRV test is a method of measuring the reaction of the autonomic nervous system. The sympathetic nervous system is activated during experiences of tension and stress, and the parasympathetic nervous system activates during relaxation. The activities of the autonomic nervous system and the sympathetic nervous system are measured by analyzing the power spectrum for the change in the interval. HF (high frequency) indicates the activity of the parasympathetic nervous system, and LF/HF (a ratio of Low Frequency to High Frequency) indicates the balance between the sympathetic and parasympathetic nervous systems. In this study, participants were measured in comfortable sitting positions for three minutes using uBioMacpa(Biosense creative, Seoul, Korea) instruments.

### 2.5. Data Analysis

All statistical analyses were performed using SPSS 21.00 (SPSS, Chicago, IL, USA). A response paired sample t-test was performed to identify changes in psychological well-being and HRV between the pre- and post-test. ANCOVA was conducted to compare differences between groups (experimental and control). The pretest results were used as covariates (baseline data) to eliminate the effects of different levels of individual psychological well-being and HRV. All statistical tests were performed at a significance level of *p* < 0.05.

## 3. Results

### 3.1. Psychological Well-Being (Well-Being Manifestation Measure Scale)

#### 3.1.1. Results of Measurement for Pre- and Post-Test Psychological Well-Being

The results of paired t-tests between pre- and post-tests, which verified how affected the psychological well-being for each group presented in Table 3. The results showed that forest therapy programs had a positive effect on the psychological well-being of juvenile probationers. The psychological well-being score of the probationary youth who participated in the forest therapy program increased significantly (*t* = −5.64, *p* = 0.000). Among the subfactors of psychological well-being, self-esteem (*t* = −4.66, *p* = 0.000), mental balance (*t* = −4.37, *p* = 0.000), social involvement (*t* = −4.31, *p* = 0.000), sociability (*t* = −3.13, *p* = 0.004), control of self and events (*t* = −5.73, *p* = 0.000), and happiness (*t* = −5.53, *p* = 0.000) showed improvement in the mean value improved and showed significant results. Conversely, the control group did not show effects on the psychological well-being of juvenile probationers. Control group did not change significantly, not only in psychological well-being, but also in subfactors such as self-esteem, mental balance, social involvement, sociability, control of self and events, and happiness.

#### 3.1.2. Results of Psychological Well-Being Measurement in Each Group

**Figure 3:**
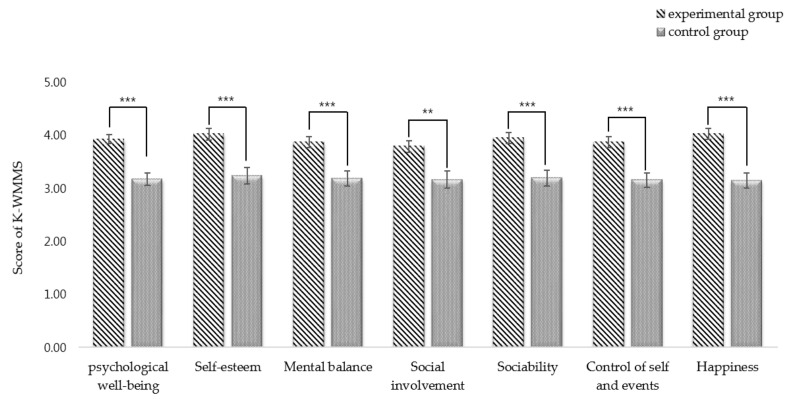
The effect of forest therapy on psychological well-being, comparing experimental and control groups, ** *p* < 0.01, *** *p* < 0.001.

To examine the effects on juvenile probationers’ psychological well-being, a covariance analysis (ANCOVA) was conducted to compare the different values before and after the program experience for each group (see Figure 3). It was confirmed that the forest therapy program resulted in positive changes in psychological well-being (F = 27.348, *p* = 0.000). In the verification of the subfactors of psychological well-being, the experimental group that attended the two-day/one-night forest therapy program showed a positive change in self-esteem (F = 18.018, *p* = 0.000), mental balance (F = 14.929, *p* = 0.000), social involvement (F = 10.163, *p* = 0.003), sociality (F = 18.019, *p* = 0.000), control of self and events (F = 17.047, *p* = 0.000), and happiness (F = 25.787, *p* = 0.000).

### 3.2. HRV

#### 3.2.1. Measurement Results for Pre- and Post-Test HRV

The results of paired t-tests between pre- and posttests, which verified how affected the HRV for each group presented in Table 4. HF and LF/HF show the activity of parasympathetic nerves, which is an indicator of HRV. The forest therapy program participation group showed positive changes in HF (*t* = −3.77, *p* = 0.001) and LF/HF (*t* = 4.38, *p* = 0.000). The juvenile probationers’ activity in the forest was physiologically stable and positively affected. Conversely, the control group did not show effects on the HF or LF/HF of juvenile probationers.

#### 3.2.2. Results of HRV Measurement in Each Group

**Figure 4:**
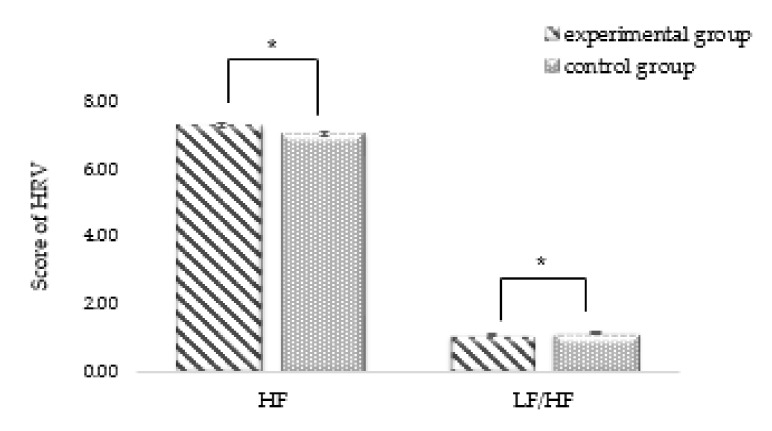
The effect of forest therapy on HRV by comparing experimental and control groups, * *p* < 0.05.

ANCOVA was conducted to compare the value differences between pre- and post-HRVs and identify physiological changes between the different program groups. As a result of HF, a positive change (F = 5.280, *p* = 0.026) was observed in adolescents who received forest therapy. In the experimental group, LF/HF also showed a positive effect (F = 4.848, *p* = 0.033) (see Figure 4). The forest environment creates comfort for the human body, and this positively affects physiological changes. It has been shown that forest therapy participants can achieve emotional stability through autonomic nervous system balance and parasympathetic nerve activation.

## 4. Discussion

This study was conducted to promote the psychological and physical stability of probationary youth by participating in a camp-type forest therapy program, which was organized to reflect their characteristics, for two days and one night. The results of this study showed that the forest therapy program influenced the psychological well-being and physical health of probationary adolescents. Empirical studies show that forest therapy programs, as well as simple activities in the forest, affect the health of participants. However, few studies have reported the psychological and physical effects of forest therapy programs for adolescents under probation. Therefore, this study suggests that these programs can help improve the psychological and physical health of probationary adolescents.

The results of the effectiveness of forest therapy programs for adolescents under probation are as follows: First, the experimental group that received the forest therapy program experienced a significant improvement in psychological well-being after the program. Self-esteem, mental balance, social involvement, sociability, control of self and events, and happiness, which are components of psychological well-being and have greatly improved. However, there were no significant changes in the control group. These results are consistent with prior studies that show that activities in forests have a positive effect on the psychological well-being [63,64,65]. Kim et al. [63] showed a change in the psychological well-being of high school students who received forest therapy programs using school forests and the attitude toward forests. You et al. [64] reported that a forest therapy program helped relieve psychological well-being, depression, and stress. Lee et al. [65] reported that forest healing programs improved participants’ psychological well-being, optimistic personal relationships, autonomy, and purpose of life. Juvenile delinquents experience more negative than positive emotions. According to a study by Shin et al. [66], the forest is a place where one can experience pleasure and self-realization by performing activities that create a sense of accomplishment, exploration, and adventure (in line with the pleasure of flow). This suggests that forests produce a restoration effect, reducing negative emotions and shifting emotional states [40,41,42]. Furthermore, active forest intervention may have a positive effect on the psychological well-being of probationary adolescents.

Second, the experimental group that received the forest therapy program experienced significant improvement in HRV’s HF and LF/HF physiological stability after the forest therapy program. However, there was no significant change in the control group. This was consistent with the findings of prior studies that forest activities provide physiological stability through the activation of parasympathetic nerves [42,46,47,48,49,67,68]. A stress is a process by which a person responds with specific physiological responses and actions to situations that threaten their well-being and health [69]. In situations of excitement and stress, sympathetic nerves are activated, increasing heart rate, and maintaining tension; it is, therefore, important to activate the parasympathetic nerves so that the body can stabilize. Woo et al. [67] reported a significant increase in parasympathetic nerves (HF) after conducting a forest therapy program for patients with depression. According to Li et al. [68], autonomic neuronal balance (LF/HF) and stress index significantly decreased after experiencing a long-term forest therapy program (six nights and seven days). Activities in the forest help stabilize the body and mind. Quintana et al. [70] reported that the social and cognitive ability to understand other people’s feelings, thoughts, etc. was reportedly superior to that of other students when the parasympathetic nerves were activated. Sensitive and nervous adolescents’ emotions can negatively affect their physical responses and are thought to have a positive effect on the physiological stability of probationary adolescents with aggressive and spontaneous characteristics.

Third, there was a clear difference between the forest therapy group and the control group. The forest experience not only positively affected the development of social emotions, such as emotional acceptance and improved emotional control of teenagers, but also provided physiological stability. Forest therapy played a role in reducing stress from everyday life. The program intervention, which combines the healing elements of nature to help subjects improve their activities in the forest, is likely to have promoted physical stability changes, increasing their resilience in the natural environment.

The forest therapy experience for probationary youth affected not only their emotional stability, but also their mental and physical resilience. Forest therapy played a role in reducing stress from everyday life. These programs may improve adolescents’ positive thinking toward the future along with an increased sensitivity or sense of cooperation with others. All these factors contribute to character development and personal growth. Such programs can play an active role in improving mental health and for reaching target goals for probationary youth. Forest therapy programs are not passive activities. Instead, participants experience the forest through meditation, physical activity, mental and physical relaxation, and ecological education. This mental health service provided to adolescents has a significant impact on delinquency behaviors and reducing recidivism [71,72]. Thus, adolescents with weak social ties and low empathy can experience changes as these programs reduce aggression and encourage group cooperation. Participation in the camp-like forest therapy program may ultimately help with social participation, social improvement, and relationships with others. Adolescence is a time when emotional instability, extreme emotions, and emotional intensity are at their highest point [73]. Considering that this is the time when emotional and physiological health is responsive to psychological stability, the forest therapy program can provide a sense of psychological relaxation. Further implementation as an attendance center orders program will ultimately have a positive effect on crime prevention and juvenile guidance.

Several limitations exist in this study. First, it is difficult to identify and present a suitable period for the effectiveness of participants in the detailed program with a program that lasted for one night and two days. Further research is needed to clarify the effects of the participation period and the continued effectiveness of the program after returning to daily life. Second, the sample size was small, and there are limitations to generalizing the results. Therefore, further research is needed for a variety of participants, depending on the type and motivation, characteristics of the individual offenses. In the case of juvenile delinquents, there are various factors that affect crime. Therefore, different types of interventions are needed. Third, participants’ experience, satisfaction, and preferences in the forest can affect the results. Due to the participants’ satisfaction with the forest, it can be difficult to see it as an effect of the forest therapy program alone. Fourth, there was minimal control over the daily activities of the control group. The types of activities other than program activity hours in the control group (i.e., academic, family, peer relations, etc.) may have varied, which may have affected the outcome comparison. Fifth, a deeper approach is needed to discuss new possibilities for the psychological and physical well-being of probationary adolescents. Additional research using qualitative analysis is needed to understand the process of changes in an individual’s emotions and cognition through forest therapy programs. In addition, longitudinal studies on the changes in participants will also be needed. These limitations should be considered for future research.

Nevertheless, this study is significant in that it verified the applicability and effectiveness of forest therapy programs with special groups of youth guardians. Future studies should organize various forms of forest therapy programs tailored to the individual needs and goals of probationary youth. It is reported that the forest therapy program will contribute to the social and emotional development of probationary youth, which is the purpose of the probation system.

## 5. Conclusions

The results of this study show that forest therapy can help improve the psychological and physiological symptoms of emotionally unstable juvenile probationers, who are establishing themselves in various experiences at this stage of life. Therefore, forest therapy can contribute to emotional stability and health promotion if it is used to manage psychological stability.

## Figures and Tables

**Table 1:** Demographic characteristics of experimental and control groups.

Classification		Experiment Group (*N* = 33)	Control Group (*N* = 17)	Total
		*N* (%)	*N* (%)	*N* (%)
Sex	Male	32 (68.1%)	15 (31.9%)	47 (100.0%)
	Female	1 (33.3%)	2 (66.7%)	3 (100.0%)
Age average	1999–2003	16.4	15.8	16.1
Academic Background	Middle school enrolled	8 (55.0%)	6 (45.0%)	14 (100.0%)
	High school enrolled	25 (71.5%)	11 (28.5%)	36 (100.0%)

**Table 2:** Forest therapy program.

Time	Day 1	Day 2
07:00-		-Get up -Morning walk
08:00–09:00		Breakfast
09:00–11:00		-Storytelling: my dream -Hammock healing -Scent of forest therapy
11:00–12:30	-Orientation and fill in the consent form -Physiological and psychological pretest	-Physiological and psychological posttest
12:30–13:30	Lunch	Lunch
13:30–14:00	Check in	
14:00–15:00	-Body, hello! (Forest gym exercise) -Introduce myself using natural objects	
15:00–15:40	Sense awakening walk	
15:40–16:20	-Dream of trees (investigating organic and dynamic ecological links of forest) -My dream (cutting logs and watching their growth rings)	
16:20–17:00	Meditation with walking in forest: slow pace	
17:00–18:00	Looking at the sky	
18:00–19:30	Dinner	
19:30–20:50	Night walk in the forest	
20:30-	Free time and off to dream land	

**Table 3:** Comparison of pre- and post-tests for psychological well-being between the experimental and control groups.

Variable	Subfactor	Experiment Group (*N* = 33)			Control Group (*N* = 17)		
		Pretest	Post-Test	t	Pretest	Post-Test	t
		M ± SD	M ± SD		M ± SD	M ± SD	
Psychological well-being	Self-esteem	3.30 ± 0.86	4.01 ± 0.71	−4.66 ***	3.40 ± 0.77	3.26 ± 0.73	2.08
	Mental balance	3.18 ± 0.83	3.85 ± 0.70	−4.37 ***	3.33 ± 0.73	3.22 ± 0.61	1.13
	Sociability	3.13 ± 0.80	3.77 ± 0.65	−4.31 **	3.34 ± 0.51	3.21 ± 0.75	1.09
	Social involvement	3.48 ± 0.89	3.96 ± 0.70	−3.13 ***	3.41 ± 0.59	3.18 ± 0.54	0.75
	Control of self and events	2.97 ± 0.88	3.83 ± 0.69	−5.73 ***	3.25 ± 0.54	3.22 ± 0.50	1.59
	Happiness	3.12 ± 1.01	3.90 ± 0.61	−5.53 ***	3.47 ± 0.58	3.24 ± 0.46	0.27
	Total	3.19 ± 0.78	3.90 ± 0.61	−5.64 ***	3.37 ± 0.46	3.22 ± 0.46	2.12

M = mean; SD = standard deviation ** *p* < 0.01 *** *p* < 0.001 by paired *t*-test.

**Table 4:** Comparison of HRV pre- and posttests between the experimental and control group.

Variable		Experiment Group (*N* = 33)			Control Group (*N* = 17)		
		Pretest	Post-Test	t	Pretest	Post-Test	t
		M ± SD	M ± SD		M ± SD	M ± SD	
HRV	HF	6.98 ± 0.64	7.32 ± 0.54	−3.77 **	6.91 ± 0.66	7.03 ± 0.31	−1.15
	LF/HF	1.18 ± 0.11	1.09 ± 0.09	4.38 ***	1.20 ± 0.13	1.15 ± 0.08	1.37

M = mean; SD = standard deviation ** *p <* 0.01, *** *p* < 0.001 by paired *t*-test.

## Data Availability

The data presented in this study are available on request from the corresponding author. The data are not publicly available due to privacy.
